# What Not to Do for Patients

**Published:** 1918-09

**Authors:** Frank W. Sage

**Affiliations:** Cincinnati, O.


### WHAT NOT TO DO FOR PATIENTS

BY FRANK W. SAGE, D.D.S., CINCINNATI, O.

The young dentist, looking back over the first few years of practice, finds numbers of instances in which he has experimented at loss to himself, for his patient's benefit. The appliance specially devised, the unusual operation performed, did well for a time only, to fail, finally. Now, perhaps, he finds himself confronting the alternative of doing the work over for a nominal fee or none at all, or offending his patient.

Dentists learn sooner or later to limit their experimenting with appliances or methods not fully tested. The dentist-inventor who glows with enthusiasm as he describes in the dental convention his new appliance or method, may be pardoned for his partiality; nevertheless, the young dentist owes it to himself to examine for himself with closest scrutiny, with a shrewd eye of forecast, before adopting the new suggestion. Many devices or suggestions will do only for exceptional cases. The dentist needs to use his eyes, his judgment, to foresee the probable outcome.

It is no difficult matter to decide whether this or that operation or appliance will do the work. Yet every dentist sees daily, operations by fellow practitioners which a little deliberation, a little forecasting of results, should have forbidden. Often, no doubt, the patient's fear, or his persistent adhearance to some preconceived notion, swerves the dentist from a better course he might have taken. It is the height of folly in the dentist to be influenced by the wilful insistence of a patient who knows nothing whatever of the requirements of his own case, and the dentist who is aware of weakness in listening, needs to take himself firmly in hand, first of all. I might dwell here at length on this point, but as Kipling says, that is another story.

Still, we must experiment if we would progress. Note here the supreme importance of judiciously selecting the patient for the experiment. The temptation is strong to try the new method or appliance in some desperate case where nothing else promises success, where even the new method is attempted with serious misgivings. The dentist in his heart of hearts feels that not even the new suggestion will do, yet in sheer desperation tries it. When failure results he perhaps rejects for all time the really valuable novelty.

Tact in handling patients means taking no risks with certain classes of people. Here comes a lady who takes your hand mirror and fussily indicates imperfections in the work you have done for her, imperfections which exist only in her imagination; telephones that fillings have dropped out, which you find in place—in short, is never quite satisfied with anything you do for her. She is the least desirable subject for an experiment, yet is the very one to set you to work on impossible undertakings, to gratify her whims.

A better subject is the portly, genial gentleman who pooh-poohs at anesthetics, who will let you stuff a napkin half way down his throat, without protest. The patient who prefers to pay you more, rather than less, when he leaves the chair. We all have a few such.

Do not be tempted to depart very far from that to which your patient has been long accustomed, especially as to the new plate or bridge. You find a plate with upper incisors shutting within the lower circle, after a natural prognathism, no doubt, and you resolve to give the patient a pleasant surprise by making the new plate she requires, with incisors outside. Ah, ah, look out! If you only knew, the other dentist tried that, and had to change the occlusion to that you find in the old plate. This is a common error. Be not too sure, in making another plate, that a new shape, size, color of the teeth, will be regarded as an improvement. Be sure you consult the patient as you go along, if you make such changes. Here comes a patient wearing a plate with teeth arranged with faultless regularity. You perceive a thinness of visage, an angularity of features arguing that her own natural incisors overlapped. You change the arrangement scheme accordingly, and are mortified to find neither she nor her friends like it.

Another patient challenges your admiration of a set of teeth she wears, which to your conception are glaringly wrong as to color, size, arrangement. Do not, as you value your reputation, go ahead with a new set radically different from the old one. You might almost as well tell some mothers their babies are not pretty, as to criticise their artificial teeth. Be careful!

This, of course, does not mean you are to repeat the error. Yet some patients are so constituted that they shut their ears to suggested improvement.

If the young dentist is aware of not ranking among the most skilled of the profession, he needs to consider very carefully whither he is likely to be led in attempting an unusual operation, especially one involving much labor and expense. As you value your time and labor, beware of insisting in a matter concerning which the patient manifests indifference, or perhaps even disapproval. Even though you are sure of being able to accomplish something which ought to surprise and gratify the patient, the question first of all arises, is this patient one of the appreciative sort? Some patients are so constituted after a perverse fashion, that they refuse to see good in even an invaluable service. With such, do not insist.

Among the things not to do for a patient, is to attempt some operation by way of correcting an evil complained of, which operation, if unsuccessful, is liable to aggravate the evil condition. For instance: a lady presents with an amalgam filling in the lingual surface of an incisor, which has stained through to the labial surface. Before attempting to remove the filling with a view to substituting cement or other filling, consider that such stain denotes an approach to the pulp more or less imminent, so that removing the filling and all the stain must mean a still nearer approach to the pulp and increasing risk of causing its death.

If your patient is one of the captious kind, it is better plainly to indicate the danger, impressing upon him or her that removal of the pulp means loss of color in the tooth. I believe it is often best, dealing with a captious patient, to resort to the more radical way, as in this instance, of crowning, to avoid a possibility of criticism on account of a change of color. Yet through unwillingness to cause the patient expense, many dentists in this instance would risk irritating the pulp, in order to restore the color with a cement filling or lining. At all events, fail not to apprise the patient of complications liable to ensue on removing the old filling. Little observances of the kind make the difference, ofttimes, between a patient lost and one placated and retained.

Since I have drifted into narration of specific cases, let me add another: do not fill over pulps liable to ache. In principle it is all very well to attempt saving every pulp, as was done years ago far more than today. In practice, the good occasionally achieved is far more than offset by the resulting complications of pain and inconvenience attending many failures. Consider for a moment: if it comes to the necessity of removing a filling and the pulp later, you have a case difficult in the extreme to manage, to wit: a congested and inflamed pulp insusceptible to the action of devitalizing agents, or obtundents; in short, a pulp which can be extirpated, nine times out of ten, only at the expense of severe pain to the patient, and prolonged after-treatment. Whereas, if the pulp had been boldly attacked in the first place, while uninflamed and uncongested, it might easily and probably painlessly have been destroyed, and the patient saved from a most unfavorable impression of dental ministrations. [Note—Healthy pulps are easily devitalized.]

I long since have adopted it as an axiom in my practice: a pulp which has once ached, or which has even caused serious uneasiness, must be extirpated. Such pulps never survive. I write this deliberately, after long observation.

There is an element of humbuggery, if you choose so to call it, which ought to be employed more, rather than less, in dental practice. Here comes a girl, white with fear, in custody, as it were, of her mother, to keep an appointment. She is slight of build, pale, anemic, utterly incapable of summoning resolution to endure any pain whatsoever. You see at a glance her teeth will prove exquisitely sensitive. She is forced into your chair by the mother.

"Now, Alice, sit right down and let the doctor do what is necessary. For shame, Alice! There, don't be a great baby!"

How admirably the mother braves it out for her. But —are you going to operate for this frightened-to-death child without even a word of reassurance? On no account. Better not operate at all, today. Now for a little humbug preliminary treatment. Well—not so distinctively humbug, either. Treat those labial cavities with silver nitrate; tell her to come again in three days for a second treatment, meanwhile using an alkaline wash at night. You may even sacrifice mamma in the interests of the patient. Intimate pleasantly that mothers do not always appreciate how sensitive some teeth are. Ask her mother how she would enjoy the operation. Never mind how or what she answers; what you are after now is to establish yourself in Alice's good graces. Wait a few days, repeating the silver nitrate treatments, or sealing into other than labial cavities the required sedative. Meanwhile the young lady is becoming accustomed to your instruments, and fears them somewhat less. We need every advantage in treating such patients.

What not to do for children is perhaps the most perplexing question of all. They must be served, yet how? It is laid down as a general principle that the dentist's treatment of children must at all events be kind and gentle. But there are other ways, not the antithesis of this. I believe it a good plan to express your displeasure with the actions of certain children. Discovering that they are simply seeking to delay and thwart your efforts, it is often an excellent way to desist, after a time, refuse to make further attempt, and dismiss the little patient. Let him see you are thoroughly displeased. Insist on his going away. Never mind the parent's insisting; dismiss the case. Later, if you please, you may indicate to the mother a willingness to try again, another day. A note discreetly worded, or a telephone message, will be likely to bring the little patient in again, wearing a chastened expression, an expression of a wish to conciliate you. This is as it should be; you have diverted the child's mind from his own fears for himself into another line of thought—a fear he may again incur your displeasure. You have thrust back on the parent responsibility for neglecting to train his or her child to "endure hardness," to submit to necessary pain. In many instances I have found this plan to work admirably.

In one instance I tried another ruse. A girl of nine, brought in by her mother, refused for an hour so much as to open her mouth for an examination. Sat dumb, flintily immovable. I learned in a little aside from her mother that she had had several teeth filled the week before, since which event it had been found impossible to persuade her to return to that dentist.

The ludicrous aspect of the situation so impressed me that I burst into laughter, greatly to Mary's amazement. However, she remained obdurate, and her mother took her away, venting her displeasure, declaring that

Mary should stay at home with the cook all summer, while the family sojourned at the seashore. I secretly indicated to her that she might hear from me presently.

That afternoon I wrote a note, not to the mother, to Mary. I intimated that we had got a wrong start that morning, delicately leading up to a suggestion that mothers are apt to be a hoodoo in a dental office, and suggesting that she come alone, at ten the next day, and I thought we might do much better. At nine the next morning came a telephone message, Mary's voice, subdued, wary. She said she would come, and "Mind, don't you tell, for nobody knows but the governess."

Sure enough, at ten in she came, radiant, smiling, and sat down in my chair without a suggestion of hesitation. I filled three teeth, and while she protested somewhat, it was a protest indicating a determination to brave it out, at all events. Shook hands at parting, and said, "Wasn't it a good joke on mamma?"

We must often resist the mother who declares she can not come again; too many engagements. If you deem it necessary, for prudential reasons, to temporize with a child, let not the mother's objections prevail. It is better to state reasons repeatedly, if required, in order suitably to impress the mother. She will think all the better of you for firmly insisting on your suggestions being carried out even at inconvenience to herself. That is a phase of human nature many dentists seem not to recognize. You must often insist, openly thrusting back upon the mother responsibility for ignoring your wishes. She will respect you all the more.

This is one of the suggestions which should not be necessary, yet are eminently necessary for the guidance of every weak-willed dentist, who needs to begin with himself in correcting errors in his practice.

In order to get a dental practice into smooth working order, numerous apparently trifling matters, concerning the relations with parents and guardians, must receive diligent attention. After a year or two, the dentist's reputation for business-like methods in appointments will have become established, and he will find he has the upper hand of his practice, instead of being at the mercy of any one who chooses to abuse his leniency.—*Dental Summary*, August, 1918.

